# Total Antioxidant Concentrations of Breastmilk—An Eye-opener to the Negligent

**DOI:** 10.3329/jhpn.v29i6.9897

**Published:** 2011-12

**Authors:** Arun Mamachan Xavier, Kavita Rai, Amitha M. Hegde

**Affiliations:** ^1^Department of Pediatric Dentistry, Amrita School of Dentistry, AIMS Ponekkara PO, Cochin, India; ^2^Department of Pedodontics and Preventive Children Dentistry, A.B. Shetty Memorial Institute of Dental Sciences, Mangalore, India

**Keywords:** Antioxidants, Breastfeeding, Breastmilk, Milk, Human, Total antioxidant capacity, India

## Abstract

The balance between free radical production and antioxidant defenses in the body has important systemic and oral health implications. There is convincing evidence that breastmilk containing antioxidants is important in the prevention of diseases in infancy. This study compared the total antioxidant concentration of human breastmilk expressed at different stages of lactation, stored at various temperatures and durations. Expressed breastmilk (EBM) samples of the third, seventh and 30th day were collected from women who had term and preterm deliveries (n=20). Another cohort of women (n=20) was also assessed; these women were more than five months postpartum and lactating. The total antioxidant capacity (TAC) of EBM was assessed at zero hours at room temperature, at 48 hours, one week post-refrigeration (4 °C), and freezing (-8 °C) respectively using the phosphomolybdenum method. The highest antioxidant levels were found in colostrum. The TAC of EBM reduced with time and at post-refrigeration and after freezing (p<0.0005). No significant difference in the mean TAC was observed between the EBM samples obtained from women with either term or preterm deliveries. The progressive loss of antioxidant content of EBM emphasizes the need of awareness and curtailment of the practice of storing and later use of EBM.

## INTRODUCTION

The period from birth to two years of age is a ‘crucial window’ for the promotion of optimal growth, health, and cognitive development of a child. Adequate nutrition through appropriate infant and young child-feeding (IYCF) practices is fundamental to the development of each child's full human potential (1). Human milk is species-specific, and all substitute feeding preparations differ remarkably from it, making human milk uniquely superior for infant feeding. It contains, on average, 1.1% protein, 4.2% fat, and 7.0% carbohydrate and supplies 72 kcal of energy per 100 g (2).

Premature infants (<37 weeks gestation) are a very vulnerable group. They are at a high risk of medical and surgical complications that are costly to the healthcare system and compromise the quality of their lives (3). There is strong evidence that feeding of human breastmilk decreases the incidence and/or severity of various infectious diseases, including bacterial meningitis (4), bacteraemia (5), diarrhoea (6), respiratory tract infection (7), otitis media (8), and late-onset sepsis in preterm infants (9).

Saugstadt suggests that all the factors, conditions, and problems affecting infants, especially those born prematurely, are the outcomes of one unifying disease—oxygen radical disease (10). If there are too many free radicals produced and too few antioxidants, a condition of oxidative stress develops, which may cause serious damage in infancy. A need to reduce oxidative stress and/or boost antioxidant defenses in these vulnerable infants is essential.

Human milk has antioxidant properties. It contains vitamin C and E and enzymes, including superoxide dismutase, catalase, and glutathione peroxidase. These are known to protect against the potentially-harmful effects of oxidative stress (3). Apart from its role in maintaining the viability and texture of human tissue cells, it also modulates immune-mediated mechanisms in the body for a healthy survival. The role of antioxidants has also been depicted in the first window of infectivity, gingival and periodontal diseases, oral infections, and tissue morphogenesis. Hence, antioxidants form an important part of diet and, together with intracellular antioxidant enzyme systems, prevent various diseases (11).

The storage of expressed breastmilk (EBM) for later use by working mothers is well on the rise. Feedingbottle systems are used for delivering stored EBM to infants (12). Evidence shows that storage of human milk and infant formula leads to a loss of ascorbic acid (12). The purpose of this study was to evaluate the influence of storage temperature and duration on the total antioxidant capacity (TAC) of human EBM.

## MATERIALS AND METHODS

The study was carried out at Justice K S Hegde Charitable Hospital, Mangalore and at the Department of Pediatric Dentistry of A.B. Shetty Memorial Institute of Dental Sciences, Mangalore, India.

### Study subjects and samples

The study women included inmates from the Gynecology Ward of Justice K S Hegde Charitable Hospital and the local residents. They were healthy women aged less than 30 years and belonged to the middle socioeconomic strata. The categorization of economic backgrounds was made based on a standard annual income chart. Mothers belonging to the middle-class families and of similar ages were selected for the study, assuming that wide discrepancies among their lifestyles and dietary patterns would not exist.

Based on a pilot study for a pooled standard deviation of 8 µg/dL in the TAC and the clinical difference of 10 µg/dL, for a power of 80%, the sample-size calculated was 14 in each experimental group. Although the research initiated with 14 mothers in a group, four mothers had to be excluded as three failed to report on the mentioned day for the collection of samples and one fell ill. To standardize the total number of samples in each group, 10 term and preterm mothers each were selected for the respective analyses. The clinical and demographic data of the selected mothers are summarized in Table 1.

### Study design

Two mL of breastmilk was collected on the third day (colostrum), seventh day (transitional milk), and 30th day (mature milk) postpartum from 10 mothers each who delivered at term (n=10) and preterm (n=10). EBM samples were also collected from randomly-selected mothers who delivered at term (n=10) and preterm (n=10) and who were five months to one year postpartum.

Breastmilk for the measurement of TAC was expressed into sterile plastic containers. The sample was then divided into five aliquots. The fresh samples were immediately tested, and the remaining aliquots were stored at 4 ºC and at minus;8 ºC for analysis at 48 hours and one week (Fig.).

**Table 1.** Clinical and demographic data of milk donors (n=40)

Parameter	No.
Age (years)	
Median	27
Range	20-30
Gestation (weeks)	
Preterm (<37 weeks)	
Median	36
Range	34-37
Term (>37 weeks)	
Median	39.75
Range	38-41
Weight (kg)	
Median	64.5
Range	55-72
Geographic location	
Rural	37
Semi-urban	3
Urban	-

### Estimation of total antioxidant capacity

The phosphomolybdenum method was used for assessing the TAC of the milk samples (13). An aliquot of 0.1 mL of the sample solution containing a reducing species (ethanol) was combined in an Eppendorf tube. To this, 1 mL of reagent solution, containing 28 mM of sodium monophosphate, 0.6 mM of sulphuric acid, and 4 mM of ammonium heptamolybdate, was added. The Eppendorf tubes were capped and incubated in a thermal block (Rotek Co., Ernakulam, India) at 95 °C for 90 minutes. The samples were then allowed to cool at room temperature. The absorbance of the aqueous solution for each sample was measured at 695 nm against a blank solution using a spectrophotometer (Spectrophotometer 106, Systronics Co., Ahmedabad). The typical blank solution contained 1 mL of reagent alone without the test solution and was incubated under the same conditions as the remaining samples.

### Analysis of data

Data were analyzed using Student's *t*-test and repeated measures (analysis of variance−ANOVA), with Bonferroni multiple comparison test. The paired *t*-test compared the variations in the TAC between two storage periods within a group while the unpaired *t*-tests compared the TAC inter-group following a particular storage period. The repeated measures ANOVA was used for statistically assessing the variations in the TAC through all the phases of lactation. The differences at the 5% level of probability were considered significant. The SPSS software (version 15.0) (SPSS Inc., Chicago, IL, USA) wasused for all analyses.

**Fig.**
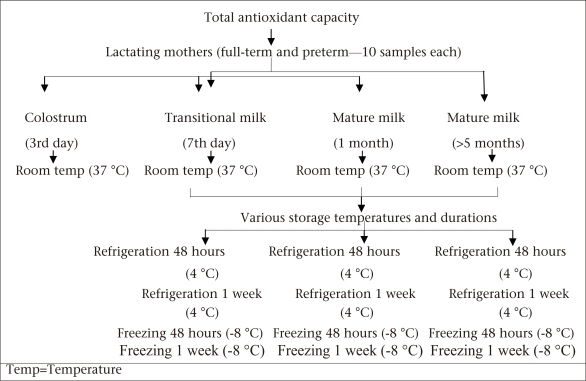
Study design

**Table 2.** Total antioxidant capacity of breastmilk assessed immediately following expression at room temperature

Lactating mothers	Mean TAC (SD) (µg dL)			Statistical significance		
	Colostrum	Transitional milk	Mature milk	Colostrum vs T. milk	Colostrum vs T. milk	T. milk vs T. milk
Mothers delivered full-term	112.9 (8.38)	103.93 (7.13)	101.82 (6.73)	8.97*	11.08**	2.11
Mothers delivered preterm	104.6 (6.02)	98.47 (6.49)	97.45 (6.71)	6.13*	7.15*	1.02

* abc

The repeated measures ANOVA and Bonferroni multiple comparison analyses were the statistical tests applied.

*p<0.05;

**p<0.005;

ANOVA=Analysis of variance;

M=Mature;

SD=Standard deviation;

T=Transitional;

TAC=Total antioxidant capacity

### Ethical aspects

The institutional committee for ethics and research approved the study protocol. A written informed consent was taken from the participants of the study.

## RESULTS

A significant difference (p<0.05) in the level of antioxidants was observed in colostrum, transitional milk, and mature milk assessed immediately following expression at room temperature (Table 2). However, Bonferroni pair-wise comparison test showed no significant difference (p>0.05) between transitional and mature breastmilk at room temperature. The TAC of colostrums in full-term and preterm pregnancies was compared. There was a significant difference (p<0.001) and higher TAC in full-term pregnancies. However, no significant difference was observed between the TAC of transitional and mature milk.

There was a significant decrease in the TAC of EBM after refrigeration and freezing for 48 hours and one week when compared with the TAC at zero hour (p<0.0005) (Table 3). This suggests that subjecting EBM to low temperatures for a prolonged time causes a significant loss of antioxidant levels. No difference was found in the TAC between transitional and mature milk refrigerated at 48 hours and one week (p>0.05).

**Table 3.** Total antioxidant capacity of breastmilk of the 7th day (transitional milk) and 30th day (mature milk) stored at various temperatures and durations

Type of milk	Mean TAC (SD) (µg/dL)				
	Room temperature 37 °C	Refrigeration 48 hours 4 °C	Refrigeration 1 week 4 °C	Freezing 48 hours minus;8 °C	Freezing 1 week minus;8 °C
Transitional milk					
Mothers delivered					
full-term	103.93 (7.13)	98.27 (7.71)	89.17 (7.41)	83.98 (6.12)	78.02 (5.23)
95% CI	98.83-109	92.7-103.7	83.9-94.5	79.6-88.3	74.2-81.7
Mothers delivered					
preterm	100.77 (6.78)	93.94 (6.3)	86.31 (5.23)	81.56 (5.67)	75.45 (4.34)
95% CI	95.9-105.6	89.4-98.4	82.5-90	77.5-85.6	72.3-78.5
Mature milk					
Mothers delivered					
full-term	101.82 (6.73)	95.99 (6.51)	89.53 (7.07)	80.89 (6.45)	76.87 (5.86)
95% CI	97-106.6	91.3-100.6	84.4-94.5	76.2-85.5	72.6-81
Mothers delivered					
preterm	97.45 (7.53)	90.12 (5.65)	89.32 (4.68)	84.45 (5.76)	74.27 (6.23)
95% CI	92.1-102.8	86.1-94.2	85.9-92.6	80.3-88.5	69.8-78.7

The paired and unpaired *t*-test analyses were applied. The TAC at all storage temperatures showed a difference from room temperature (baseline), which was highly significant (p<0.005).

CI=Confidence interval;

SD=Standard deviation;

TAC=Total antioxidant capacity

**Table 4.** Total antioxidant capacity of breastmilk from mothers following the 5th month postconception stored at various temperatures and durations (baseline to different storage temperatures and durations)

Mature milk (>5 months)	Mean TAC (SD) (µg/dL)				
	Room temperature 37 °C	Refrigeration 48 hours 4 °C	Refrigeration 1 week 4 °C	Freezing 48 hours minus;8 °C	Freezing 1 week minus;8 °C
Mothers delivered					
full-term	109.34 (11.4)	101.9 (12.03)	94.78 (11.1)	81.2 (4.76)	75.02 (5.2)
95% CI	101.2-117.5	93.3-110.5	87-102.7	77.8-84.6	71.3-78.7
Mothers delivered					
preterm	106.36 (4.23)	100.03 (5.12)	93.31 (7.16)	82.72 (4.9)	73.55 (6.39)
95% CI	103.3-109.4	96.4-103.6	88.2-98.4	79.2-86.2	68.9-78.1

The paired and unpaired *t*-test analyses were applied. The TAC at all storage temperatures showed a difference from room temperature (baseline), which was highly significant (p<0.005).

CI=Confidence interval;

SD=Standard deviation;

TAC=Total antioxidant capacity

Mature milk from the cohort of women who weremore than five months postpartum showed a similar pattern of variations in the TAC following storage at different temperatures compared to room temperature (p<0.0005) (Table 4). No statistical difference was observed between the TAC of breastmilk samples at room temperature, obtained from the full-term and preterm mothers. Table 4 illustrates the TAC levels of maternal milk following the fifth month postpartum subjected to various temperature treatments.

## DISCUSSION

Low levels of antioxidants, or inhibition of the antioxidant enzymes, cause oxidative stress and may damage or kill viable cells (14).

Breastmilk appears to be beneficial in providing antioxidative protection. The complete list of active antioxidant components in breastmilk is still unknown. Compared to infants born full-term, preterm infants are more vulnerable to oxidative stress caused by infections, mechanical ventilation, intravenous nutrition, and deficiency in alpha-tocopherol which accumulates mainly during the third trimester of pregnancy (15). The antioxidant enzyme systems contained in maternal milk help the premature infant cope with reactive oxygen species (ROS)-mediated diseases (3,16,17). Lactoferrin, abundant in human milk, is a member of the iron-binding transferrin protein family that inhibits the formation of ROS by binding iron, thereby attenuating the conversion of hydrogen peroxide into hydroxyl radical via the Fenton type reaction (18).

The ROS and antioxidant systems act in concert rather than alone (19). Hence, investigations of any individual antioxidant activity may be misleading, and the measurement of any individual antioxidant may be less representative of the whole antioxidant status. Moreover, the number of different antioxidants makes it difficult, and also expensive, to measure each antioxidant separately, especially during daily clinical treatments. For these reasons, research is now being directed towards assays that evaluate the TAC of biological fluids (20). Hence, the present study aimed at estimating the TAC of breastmilk.

In the present study, a significant difference in the TAC was observed over the three phases of milk transition (Table 2). The variations observed in the mean TAC values may be attributed to the discrepancy in the dietary patterns of the participants, the immune capacity of their bodies, and ethnic or racial differences (21,22). Thus, these values cannot be deemed universal. The TAC in colostrum from term pregnancies was higher compared to preterm pregnancies, which could probably be attributed to the increased oxidative stress; this would, however, require further investigations.

Expression and storage of breastmilk are a way to maintain breastfeeding when the mother and the infant are separated, provided its nutritional value can be conserved. The effect of storage on various components of human milk has been studied. Most of these studies have focused, however, on the bacteriological, nutritional and immunological effects of storage (23-26). Refrigeration and freezing of breastmilk cause a significant decline in the levels of individual antioxidants, including vitamin C, A, and E. Nevertheless, the values of all nutrients were still within the international reference ranges for mature breastmilk (12). The paucity of researchbased data documenting the TAC of maternal milk subjected to storage for longer durations at reduced temperatures prompted us to conduct this work.

The TAC of transitional and mature milk from both full-term and preterm mothers was subjected to refrigeration and freezing for 48 hours and one week in the present study. It was found to deplete the TAC of milk by 10-20% and 15-30% following a 48-hour and one-week storage period respectively (p<0.0005) when compared with the TAC at zero hour at room temperature (Table 3). The values obtained were similar to those found by Hanna *et al*., which compared the antioxidant capacity of breastmilk stored at lower temperatures using ABTS assay and showing the rapid decline over time, more gravely observed with the frozen samples over a seven-day period (27). Miranda *et al.* found an increase of malondialdehyde, a marker of oxidative stress, in refrigerated milk but not in frozen samples and a decrease of glutathione peroxidase activity in both refrigerated and frozen samples of human milk (28). Results of a study on the glutathione status of stored human breastmilk by Ankrah *et al.* showed a substantial loss of glutathione when breastmilk was kept at minus;20 °C, 4 °C, or at room temperature for two hours when compared with fresh breastmilk (29). Our study proved that subjecting EBM to reduced temperatures for prolonged duration significantly reduces the TAC. With the increasing number of working mothers, it is essential that they are educated by sharing with them pertinent information on the safe-handling procedures of breastmilk and its potential influence on its TAC.

Many components of milk change with storage, including immune cells, which get inactivated by freezing (24,30). A conspicuous formation of lipid peroxides in human milk stored at lower temperature is documented, probably caused by an increased presence of free fatty acids due to lipoprotein lipase activity during storage (3,31). This could be ascribed to the higher susceptibility of human milk to degradation in the current analysis, resulting in a loss of TAC over a longer storage time at reduced temperatures.

Further, long-term research can be directed towards analyzing the health effects upon feeding infants with stored milk at reduced temperatures. These scientific facts should evoke responsibility among paediatric health professionals in providing prenatal and postnatal counselling to mothers through effective strategies. With the growing concerns in today's society over maintaining a healthy lifestyle, one of the keys to protection against oxygen radical disease could be rethinking what the infant should be fed each day. Paediatric health specialists should include in their preventive strategies provision for giving antioxidants to infants through breastfeeding, emphasizing its role in health and disease.

## ACKNOWLEDGEMENTS

The authors extend their deepest gratitude to Prof. (Dr.) Suchetha Shetty, Department of Biochemistry, K S Hegde Medical Academy, Mangalore, for her sincere contributions to this research. They gratefully acknowledge the valuable assistance received from Ms Ramitha for laboratory service and Ms Neevan D'Souza for her sincere help in analyzing the study data.
